# A nomogram based on pretreatment levels of serum bilirubin and total bile acid levels predicts survival in colorectal cancer patients

**DOI:** 10.1186/s12885-021-07805-9

**Published:** 2021-01-21

**Authors:** Yinghao Cao, Shenghe Deng, Lizhao Yan, Junnan Gu, Jia Yang, Ming Yang, Li Liu, Kailin Cai

**Affiliations:** 1 Department of Gastrointestinal Surgery, Union Hospital, Tongji Medical College, Huazhong University of Science and Technology, 1277 JieFang Avenue, Wuhan, 430022 Hubei China; 2 Department of Gastrointestinal Surgery, The Central Hospital of Wuhan, Tongji Medical College, Huazhong University of Science and Technology, Wuhan, China; 3 Department of Pathology, Union Hospital, Tongji Medical, Huazhong University of Science and Technology, Wuhan, 430022 Hubei China; 4 Department of Epidemiology and Biostatistics, the Ministry of Education Key Lab of Environment and Health, School of Public Health, Tongji Medical College, Huazhong University of Science and Technology, Wuhan, Hubei 430022 China

**Keywords:** Colorectal cancer, Total bilirubin, Direct bilirubin, Total bile acid, Survival analysis

## Abstract

**Background:**

Serum bilirubin and total bile acid (TBA) levels have been reported to be strongly associated with the risk and prognosis of certain cancers. Here, we aimed to investigate the effects of pretreatment levels of serum bilirubin and bile acids on the prognosis of patients with colorectal cancer (CRC).

**Methods:**

A retrospective cohort of 1474 patients with CRC who underwent surgical resection between January 2015 and December 2017 was included in the study. Survival analysis was used to evaluate the predictive value of pretreatment levels of bilirubin and bile acids. X-Tile software was used to identify optimal cut-off values for total bilirubin (TBIL), direct bilirubin (DBIL) and TBA in terms of overall survival (OS) and disease-free survival (DFS).

**Results:**

DBIL, TBIL, and TBA were validated as significant prognostic factors by univariate Cox regression analysis for both 3-year OS and DFS. Multivariate Cox regression analyses confirmed that high DBIL, TBIL and TBA levels were independent prognostic factors for both OS (HR: 0.435, 95% CI: 0.299–0.637, *P* < 0.001; HR: 0.436, 95% CI: 0.329–0.578, *P* < 0.001; HR: 0.206, 95% CI: 0.124–0.341, *P* < 0.001, respectively) and DFS (HR: 0.583, 95% CI: 0.391–0.871, *P* = 0.008; HR:0.437,95% CI: 0.292–0.655, *P* <0.001; HR: 0.634, 95% CI: 0.465–0.865, *P* = 0.004, respectively). In addition, nomograms for OS and DFS were established according to all significant factors, and the c-indexes were 0.819 (95% CI: 0.806–0.832) and 0.835 (95% CI: 0.822–0.849), respectively.

**Conclusions:**

TBIL, DBIL and TBA levels are independent prognostic factors in colorectal cancer patients. The nomograms based on OS and DFS can be used as a practical model for evaluating the prognosis of CRC patients.

## Background

CRC ranked third among the three most common cancers in both males and females in 2019 and is one of the leading causes of cancer-related mortality worldwide. It is estimated that as of January 1, 2019, more than 1.5 million men and women in the United States had been diagnosed with colorectal cancer, and 145,600 new cases will be confirmed in 2019 [1]. With the improvement in living conditions and changes in dietary habits, the incidence of CRC has been increasing in China in recent years, and surgical resection is still the only treatment for CRC at present [2].

Predicting the long-term survival of patients with CRC can be challenging due to genetic, dietary, and geographical differences. However, accurate prediction of prognosis is essential for treatment selection and communication between doctors and patients. Previous studies have shown that age, lymph node status, systemic inflammation, perineural invasion, etc. risk factors can predict the survival rate of CRC patients after surgical resection, and the most important risk factor is the tumour-node-metastasis (TNM) staging system [3, 4]. However, CRC is a heterogeneous disease, and even when patients are at the same stage of disease, the prognosis varies. The current TNM staging system has the limitations of simplicity and unity and does not take into account some important variables that may affect CRC patient survival, including clinicopathological features and adjuvant therapy. Therefore, it cannot accurately predict the prognosis of CRC patients.

Serum bilirubin is the end product of haem metabolism and was once considered to have no physiological function, but the latest research has shown that it not only has many protective properties, including effective antioxidant, anti-inflammatory and anticancer activities, but is also negatively associated with the risk of a variety of cancers, including breast cancer, lung cancer, and CRC [5–8]. Additionally, abnormally high levels of TBA, which connect the intestinal microbiota with the liver and intestinal metabolism, trigger excessive harmful effects on the colonic mucosa, markedly promoting CRC progression [9]. Gao et al. suggested that increased direct serum bilirubin levels were associated with lymph node metastasis and poor prognosis in rectal cancer patients, and Zhang et al. used nomograms based on direct serum bilirubin levels to predict the prognosis of stage II and III CRC patients [5, 10]. Although considering these factors improves the prognosis of CRC, no measurement or indicator combining serum bilirubin and TBA levels has been developed. The purpose of this study was to evaluate the ability of combined pretreatment levels of serum bilirubin and TBA levels to predict survival outcomes in CRC patients after radical resection. In addition, we developed nomograms to evaluate the predictive value of pretreatment levels of serum bilirubin and TBA levels in these patients.

## Methods

### Study design and patient selection

A total of 1474 patients with colorectal cancer who underwent surgical resection at Wuhan Union Medical College Hospital between January 2015 and December 2017 were included in this study. The inclusion criteria were as follows: (1) age > 20 years old with no preoperative antitumour therapy; (2) radical resection of primary CRC; (3) pathology confirming all stage I to IV patients; and (4) complete clinical and pathological data. Patients with the following conditions were excluded from the study: (1) colonic perforation and peritonitis; (2) history of tumour and death of other causes during the follow-up period; (3) severe cardiovascular disease; (4) patients with primary hepatobiliary disease whose serum bilirubin may be increased or decreased; and (5) other interventions, such as stent placement before radical surgery.

The following parameters were included in the analysis: age, sex, smoking status, tumour history, intestinal obstruction, tumour differentiation, tumour size, tumour location, tumour T stage, tumour N stage, tumour TNM stage, perineural invasion, vascular invasion, chemotherapy, TBIL, DBIL and TBA. All patients were reclassified according to the 7th edition of the American Joint Committee on Cancer (AJCC) TNM classification, and patients with stage II disease of high risk and/or above received adjuvant chemotherapy postoperatively.

Follow-up data were obtained every three months, and when we suspected recurrence, gastroscopy and imaging were performed at that visit time. Overall survival rate (OS) was defined as the time interval from surgery to death or the last follow-up. Disease-free survival (DFS) was defined from the date of definitive surgery to the date of first recurrence (local or distant) or date of last follow-up.

### Statistical analysis

Statistical analysis was performed using SPSS 23.0 (SPSS Inc., Chicago, IL, USA) and R 4.0.0 software (Institute for Statistics and Mathematics, Vienna, Austria). X-tile 3.6.1 (Yale University, New Haven, CT, USA) was used to determine the optimal cut-off value for TBIL, DBIL, and TBA levels. The difference between the high and low level groups was evaluated by either the χ2 test or the mann-whitney U test. The 3-year OS and DFS were estimated by the Kaplan-Meier method, and the difference of variables was compared using log-rank tests. Univariate and multivariate Cox proportional hazards regression models were used to evaluate the prognostic factors for OS and DFS by calculating hazard ratios and their 95% CIs. A nomogram of the important factors related to the OS and DFS were constructed with R software, and the performance of the nomogram was evaluated by the Harrell consistency index (c-index). To further evaluate the accuracy of the nomogram in predicting prognosis, a calibration curve was generated comparing the observed results with the predicted results. A value of *P* < 0.05 was considered significant.

## Results

### Patient clinical characteristics

A total of 1474 CRC patients were recruited for this study, including 867 males and 607 females. The mean age of patients was 57.99 ± 12.26 years (range, 20–85). The median values of TBIL, DBIL, and TBA were 7.6 (range 0.20–75.30) μmol/l, 6.7 (range 0.60–50.20) μmol/l, and 4.1 (range 0.20–86.20) μmol/L, respectively.

### Associations among TBIL, DBIL and TBA levels and clinical characteristics

**Fig. 1:**
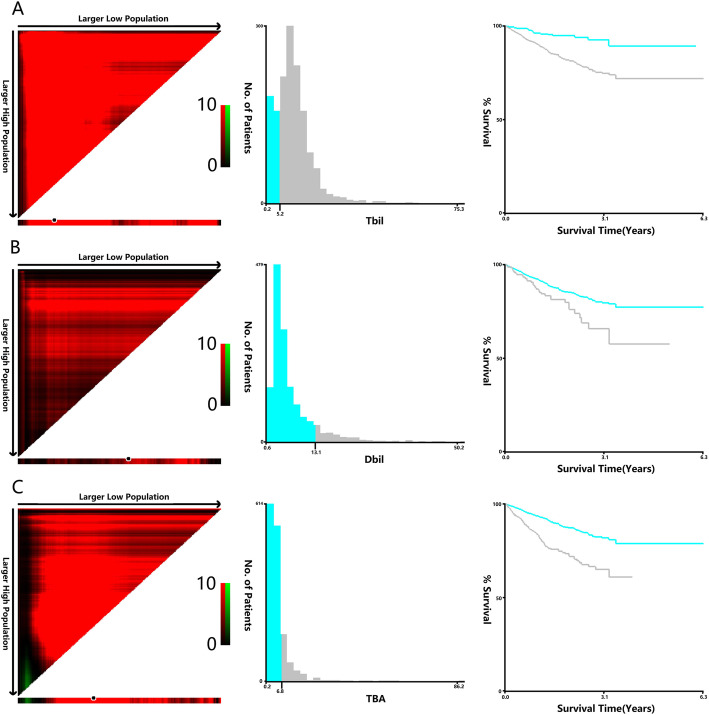
X-tile analyses of 3-year OS was performed using patients’ data to determine the optimal cut-off value for TBIL, DBIL, and TBA. X-tile analyses of TBIL (**a**), DBIL (**b**), and TBA (**c**) levels in CRC patients. X-tile plots for patients are shown in the left panels; black circles highlight the optimal cutoff values, which are also shown in histograms (middle panels). Kaplan-Meier plots are presented in right panels, and in terms of OS, the best cut-off values of TBIL, DBIL and TBA are 6.4 μmol/l, 12.8 μmol/l and 7.1 μmol/l, respectively

**Fig. 2:**
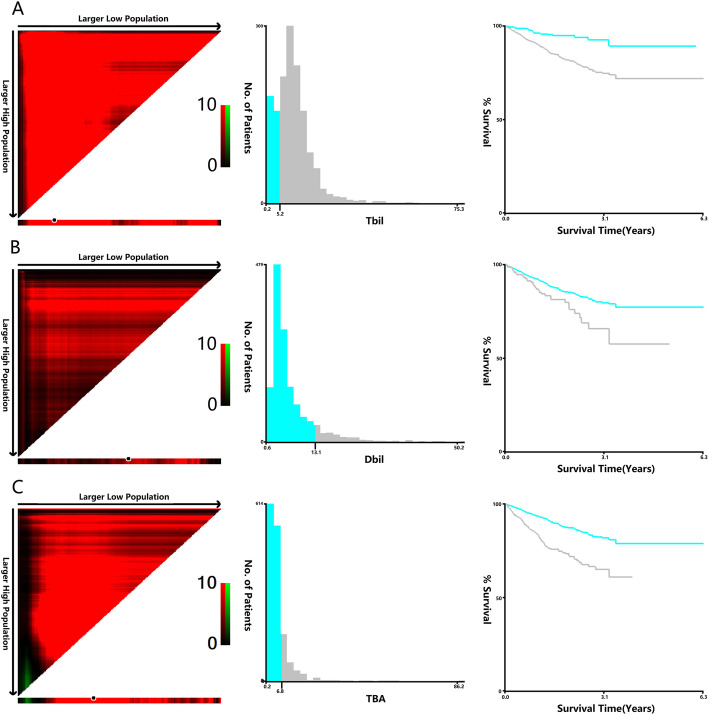
X-tile analyses of 3-year DFS was performed using patients’ data to determine the optimal cut-off value for TBIL, DBIL, and TBA. X-tile analyses of TBIL (**a**), DBIL (**b**), and TBA (**c**) levels in CRC patients. X-tile plots for patients are shown in the left panels; black circles highlight the optimal cutoff values, which are also shown in histograms (middle panels). Kaplan-Meier plots are presented in right panels, and in terms of DFS, the best cut-off values of TBIL, DBIL and TBA are 5.2 μmol/l, 13.1 μmol/l and 6.8 μmol/l, respectively

Univariate Cox regression indicated that TBIL, DBIL and TBA (when they were continuous variables) were important prognostic factors for OS and DFS, and the X-Tile program was used to determine the optimal cut-off values for DBIL in terms of OS and DFS. The optimal cut-off values were 6.4 μmol/L for TBIL, 12.8 μmol/L for DBIL, and 7.1 μmol/L for TBA based on OS (Fig. 1), and the optimal cut-off values were 5.2 μmol/L for TBIL, 13.1 μmol/L for DBIL, and 6.8 μmol/L for TBA based on DFS (Fig. 2). Next, patients were divided into high and low groups according to the optimal cut-offs. Lymph node metastasis (stage N1 and N2) was more common in patients with high TBIL than in patients with low TBIL (44.9% vs 37.4%, *P* = 0.026), but the average tumour diameter was smaller in the high group than in the low group (4.09 ± 2.11 vs 4.42 ± 2.02, *P* = 0.008) (Table 1). Compared to the low DBIL group, the high DBIL group had significantly more patients with stage III and IV disease (58.3% vs 51.8%, *P* = 0.022). In addition, males (67.5% vs 57.7%, *P* = 0.017), obstructions (22.1% vs 15.4%, *P* = 0.029), and smaller tumour diameters (3.73 ± 1.76 vs 4.25 ± 2.12, *P* = 0.003) were more common characteristics in patients with high DBIL (Table 2). Patients with stage III and stage IV disease were more commonly in the high TBA group than in the low TBA group (57.4% vs 51.5%, *P* = 0.007), and compared to the low TBA group, the high TBA group had significantly more male patients (64.4% vs 57.6%, *P* = 0.045) (Table 3).

**Table 1:** Associations between TBIL level and clinical characteristics in CRC patients

Variable		TBIL group		Statistics	P
		Low DBIL (n), %	High DBIL (n), %		
Sex	Male	239 (56.5)	628 (59.8)	1.316	0.251
	Femal	184 (43.5)	423 (40.2)		
Age		57.68 ± 12.51	58.23 ± 12.05	−0.776	0.438
Somking	Yes	91 (21.5)	212 (20.2)	0.332	0.564
	No	332 (778.5)	839 (79.8)		
Tumor history	Yes	43 (10.2)	113 (10.8)	0.11	0.741
	No	380 (79.8)	938 (89.2)		
Obstruction	Yes	68 (16.1)	170 (16.2)	0.002	0.963
	No	355 (83.9)	881 (83.8)		
Tumor size (cm)		4.42 ± 2.02	4.09 ± 2.11	2.641	0.008
Tumor location	Right colon	138 (32.6)	313 (29.8)	2.791	0.248
	Left colon	88 (20.8)	198 (18.8)		
	Rectum	197 (46.6)	540 (51.4)		
Differentiation (%)	Well	56 (13.2)	165 (15.7)	1.445	0.486
	Moderate	339 (80.1)	820 (78.0)		
	Poor	28 (6.6)	66 (6.3)		
T stage (%)	T1	36 (13.3)	72 (6.9)	3.926	0.27
	T2	70 (16.5)	169 (16.1)		
	T3	232 (54.8)	553 (52.6)		
	T4	85 (20.1)	257 (24.5)		
N stage (%)	N0	265 (62.6)	579 (55.1)	7.338	0.026
	N1	91 (21.5)	284 (27.0)		
	N2	67 (15.8)	188 (17.9)		
TNM stage (%)	I	58 (13.7)	139 (13.2)	2.973	0.096
	II	155 (36.6)	347 (33.0)		
	III	162 (38.3)	418 (39.8)		
	IV	48 (11.3)	147 (14.0)		
Perineural invasion(%)	Yes	94 (22.2)	225 (21.4)	0.118	0.731
	No	329 (77.8)	826 (78.6)		
Vascular invasion (%)	Yes	77 (18.2)	188 (17.9)	0.02	0.887
	No	346 (81.8)	863 (82.1)		
Chemotherapy (%)	Yes	226 (18.2)	556 (52.9)	0.033	0.855
	No	197 (46.6)	495 (47.1)		

*TBIL* total bilirubin, *CRC* Colorectal Cancer

**Table 2:** Associations between DBIL level and clinical characteristics in CRC patients

Variable		DBIL group		Statistics	P
		Low DBIL (n), %	High DBIL (n), %		
Sex	Male	757 (57.7)	110 (67.5)	5.681	0.017
	Femal	554 (42.3)	53 (32.5)		
Age		58.11 ± 12.16	54.74 ± 12.38	0.37	0.711
Somking	Yes	269 (28.1)	34 (20.8)	0.01	0.919
	No	1042 (71.9)	129 (79.2)		
Tumor history	Yes	139 (10.6)	17 (10.4)	0.005	0.946
	No	1172 (89.4)	146 (79.6)		
Obstruction	Yes	202 (15.4)	36 (22.1)	4.775	0.029
	No	1109 (84.6)	127 (77.9)		
Tumor size (cm)		4.25 ± 2.12	3.73 ± 1.76	2.98	0.003
Tumor location	Right colon	398 (30.3)	53 (32.5)	0.321	0.852
	Left colon	255 (19.5)	31 (19.0)		
	Rectum	658 (50.2)	79 (48.5)		
Differentiation (%)	Well	197 (15.0)	24 (14.7)	3.455	0.178
	Moderate	1025 (78.2)	134 (82.2)		
	Poor	89 (6.8)	5 (3.1)		
T stage (%)	T1	93 (7.1)	15 (9.2)	5.538	0.136
	T2	204 (15.5)	35 (21.5)		
	T3	709 (54.1)	76 (46.6)		
	T4	305 (23.3)	37 (22.7)		
N stage (%)	N0	755 (57.6)	89 (54.6)	0.81	0.667
	N1	333 (25.4)	42 (25.7)		
	N2	223 (17.0)	32 (19.7)		
TNM stage (%)	I	168 (12.9)	29 (17.8)	9.65	0.022
	II	463 (35.3)	39 (23.9)		
	III	511 (38.9)	69 (42.3)		
	IV	169 (12.9)	26 (16.0)		
Perineural invasion (%)	Yes	280 (21.4)	39 (23.9)	0.564	0.453
	No	1031 (78.6)	124 (76.1)		
Vascular invasion (%)	Yes	233 (17.7)	32 (19.6)	0.34	0.56
	No	1078 (82.3)	131 (80.4)		
Chemotherapy (%)	Yes	703 (53.6)	79 (48.5)	1.548	0.213
	No	608 (46.4)	84 (51.5)		

*DBIL* Direct Bilirubin, *CRC* Colorectal Cancer

**Table 3:** Associations between TBA level and clinical characteristics in CRC patients

Variable		TBA group		Statistics	P
		Low TBA (n), %	High TBA (n), %		
Sex	Male	699 (57.6)	168 (64.4)	4.031	0.045
	Femal	514 (42.4)	93 (35.6)		
Age		58.11 ± 12.14	57.87 ± 12.41	0.287	0.774
Somking	Yes	246 (20.3)	57 (21.8)	0.32	0.572
	No	967 (79.7)	204 (78.2)		
Tumor history	Yes	134 (11.1)	22 (8.4)	1.555	0.212
	No	1079 (88.9)	239 (91.6)		
Obstruction	Obstruction			1.617	0.203
	Yes	189 (15.6)	49 (18.8)		
	No	1024 (84.4)	212 (81.2)		
Tumor size (cm)		4.22 ± 2.12	4.07 ± 1.93	1.068	0.286
Tumor location	Right colon	363 (29.9)	88 (33.7)	1.893	0.388
	Left colon	234 (19.3)	52 (19.9)		
	Rectum	616 (50.8)	121 (46.4)		
Differentiation (%)	Well	176 (14.5)	45 (17.2)	2.653	0.265
	Moderate	955 (78.7)	204 (78.2)		
	Poor	82 (6.8)	12 (4.6)		
T stage (%)	T1	92 (7.6)	16 (6.1)	6.484	0.09
	T2	204 (16.8)	35 (13.4)		
	T3	650 (53.6)	135 (51.7)		
	T4	267 (22.0)	75 (28.7)		
N stage (%)	N0	703 (58.0)	141 (54.0)	1.449	0.484
	N1	305 (25.1)	70 (26.8)		
	N2	205 (16.9)	50 (19.2)		
TNM stage (%)	I	170 (14.0)	27 (10.3)	12.202	0.007
	II	418 (34.5)	84 (32.2)		
	III	481 (39.7)	99 (37.9)		
	IV	144 (11.8)	51 (19.5)		
Perineural invasion (%)	Yes	264 (21.8)	55 (21.1)	0.061	0.806
	No	949 (78.2)	206 (78.9)		
Vascular invasion (%)	Yes	213 (17.6)	52 (19.9)	0.814	0.367
	No	1000 (82.4)	209 (80.1)		
Chemotherapy (%)	Yes	657 (54.2)	125 (47.9)	3.391	0.066
	No	556 (45.8)	136 (52.1)		

*TBA* Total Bile Acid, *CRC* Colorectal Cancer

### Prognostic value of TBIL, DBIL and TBA levels

Patients in the smoking group (*P* = 0.002), with obstruction (*P* < 0.001), colon cancer (*P* = 0.002), advanced T stage (*P* < 0.001) and N stage (*P* < 0.001), without chemotherapy (*P* < 0.001), and with high TBIL (*P* < 0.001), DBIL (*P* < 0.001), and TBA (*P* < 0.001) exhibited a worse 3-year OS (Table 4). In addition, patients in the smoking group (*P* < 0.001), with obstruction (*P* < 0.001), left colon cancer (*P* = 0.009), advanced T stage (*P* < 0.001) and N stage (*P* < 0.001), without chemotherapy (*P* < 0.001), with high TBIL (*P* < 0.001), DBIL (*P* = 0.001), and TBA (*P* < 0.001) experienced a shorter 3-year DFS (Table 5).

**Table 4:** Univariate and multivariate analyses of OS significance of serum bilirubin and TBA levels

Variable	Univariate analysis			Multivariate analysis		
	HR	95% CI	P	HR	95% CI	P
Sex	1.065	0.823–0.232	0.630			
Age	0.994	0.984–1.004	0.268			
Somking	1.843	1.257–2.703	0.002	1.651	1.117–2.439	0.012
Tumor history	0.703	0.435–1.137	0.703			
Obstruction	2.456	1.861–3.241	< 0.001	1.843	1.374–2.473	< 0.001
Tumor size	1.01	0.951–1.072	0.748			
Tumor location						
Rectum		Reference			Reference	
Right colon	1.574	1.18–2.101	0.002	1.146	0.811–1.619	0.440
Left colon	1.56	1.12–2.171	0.008	1.400	0.603–1.111	0.199
Differentiation						
Well		Reference				
Moderate	0.99	0.699–1.402	0.954			
Poor	0.646	0.322–1.296	0.218			
T stage						
T1		Reference			Reference	
T2	0.875	0.375–2.045	0.758	0.87	0.368–2.058	0.752
T3	1.803	0.877–3.705	0.109	1.775	0.828–3.805	0.140
T4	5.968	2.915–12.219	< 0.001	3.486	1.622–7.534	0.001
N stage						
N0		Reference			Reference	
N1	2.068	1.512–2.829	< 0.001	1.311	0.861–1.998	0.207
N2	4.149	3.063–5.620	< 0.001	2.309	1.539–3.466	< 0.001
TNM stage						
I		Reference			Reference	
II	1.914	0.935–3.914	0.076	0.751	0.328–1.719	0.498
III	4.312	2.184–8.513	< 0.001	1.031	0.446–2.384	0.943
IV	10.482	5.259–20.893	< 0.001	2.588	1.135–5.902	0.024
Perineural invasion	0.869	0.632–1.196	0.390			
Vascular invasion	0.952	0.675–1.343	0.781			
Chemotherapy	0.626	0.483–0.810	< 0.001	0.579	0.442–0.76	< 0.001
TBIL	0.188	0.116–0.303	< 0.001	0.436	0.329–0.578	< 0.001
DBIL	0.528	0.376–0.742	< 0.001	0.435	0.299–0.637	< 0.001
TBA	0.26	0.201–0.337	< 0.001	0.206	0.124–0.341	< 0.001

*OS* Overall Survival, *HR* Hazard Ratio, *CI* Confidence Interval, *TBIL* Total Bilirubin, *DBIL* Direct Bilirubin, *TBA* Total Bile Acid

**Table 5:** Univariate and multivariate analyses of DFS significance of serum bilirubin and TBA levels

Variable	Univariate analysis			Multivariate analysis		
	HR	95% CI	P	HR	95% CI	P
Sex	0.928	0.711–1.212	0.583			
Age	0.995	0.985–1.006	0.379			
Somking	2.322	1.495–3.607	< 0.001	1.851	1.184–2.894	0.007
Tumor history	0.949	0.605–1.488	0.819			
Obstruction	0.504	0.375–0.678	< 0.001	0.576	0.423–0.786	< 0.001
Tumor size	0.990	0.928–1.055	0.750			
Tumor location						
Rectum		Reference			Reference	
Right colon	0.880	0.614–1.263	0.489	1.191	0.871–1.629	0.275
Left colon	0.672	0.499–0.904	0.009	1.333	0.915–1.942	0.135
Differentiation						
Well		Reference				
Moderate	1.284	0.862–1.913	0.219			
Poor	1.288	0.688–2.413	0.428			
T stage						0.004
T1		Reference			Reference	
T2	0.128	0.052–0.314	< 0.001	1.603	0.58–4.429	0.363
T3	0.177	0.105–0.3	< 0.001	2.361	0.94–5.932	0.067
T4	0.357	0.27–0.471	< 0.001	3.431	1.353–8.704	0.009
N stage						0.008
N0		Reference			Reference	
N1	1.906	1.37–2.651	< 0.001	1.120	0.75–1.673	0.580
N2	4.020	2.941–5.495	< 0.001	1.696	1.167–2.466	0.006
TNM stage						< 0.001
I		Reference			Reference	
II	2.242	0.87–5.778	0.095	1.180	0.418–3.33	0.754
III	4.968	2.008–12.295	0.001	1.980	0.713–5.497	0.190
IV	28.627	11.687–70.12	< 0.001	10.452	3.842–28.433	< 0.001
Perineural invasion	1.343	0.976–1.846	0.070			
Vascular invasion	1.165	0.826–1.643	0.385			
Chemotherapy	0.950	0.729–1.237	0.702			
TBIL	0.390	0.267–0.57	< 0.001	0.437	0.292–0.655	< 0.001
DBIL	0.618	0.429–0.891	0.010	0.583	0.391–0.871	0.008
TBA	0.408	0.308–0.541	< 0.001	0.634	0.465–0.865	0.004

*DFS* Disease Free Survival, *HR* Hazard Ratio, *CI* Confidence Interval, *TBIL* Total Bilirubin, *DBIL* Direct Bilirubin, *TBA* Total Bile Acid

Next, multivariate Cox regression analysis was performed on the clinical features that were significant in the univariate log-rank test. Results confirmed that TBIL (hazard ratio (HR): 0.436, confidence interval (CI): 0.329–0.578, *P* < 0.001; HR: 0.437, CI: 0.292–0.655, *P* <0.001), DBIL (HR: 0.4335, CI: 0.299–0.637, *P* < 0.001; HR: 0.583, CI: 0.391–0.871, *P* = 0.008), and TBA (HR: 0.206, CI: 0.124–0.341, *P* < 0.001; HR: 0.634, CI: 0.465–0.865, *P* = 0.004) levels were independent prognostic factors for both OS and DFS in patients with CRC after surgical resection (Tables 4 and 5).

### Nomogram for predicting CRC outcomes

**Fig. 3:**
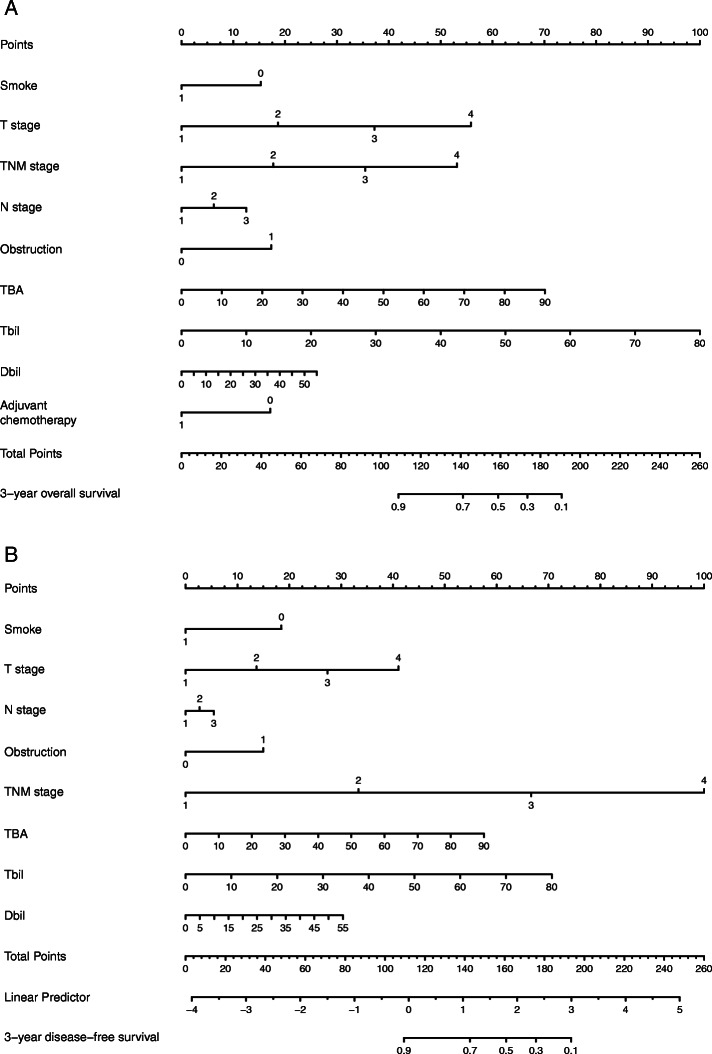
Nomogram for predicting CRC patient outcomes. Nomograms conveyed the results of prognostic models using clinicopathological characteristics and pretreatment inflammatory biomarkers to predict OS (**a**) and DFS (**b**) of patients with CRC. The Harrell’s c-indexes for OS and DFS prediction were 0.819 (95% CI: 0.806–0.832) and 0.835 (95% CI: 0.822–0.849), respectively

**Fig. 4:**
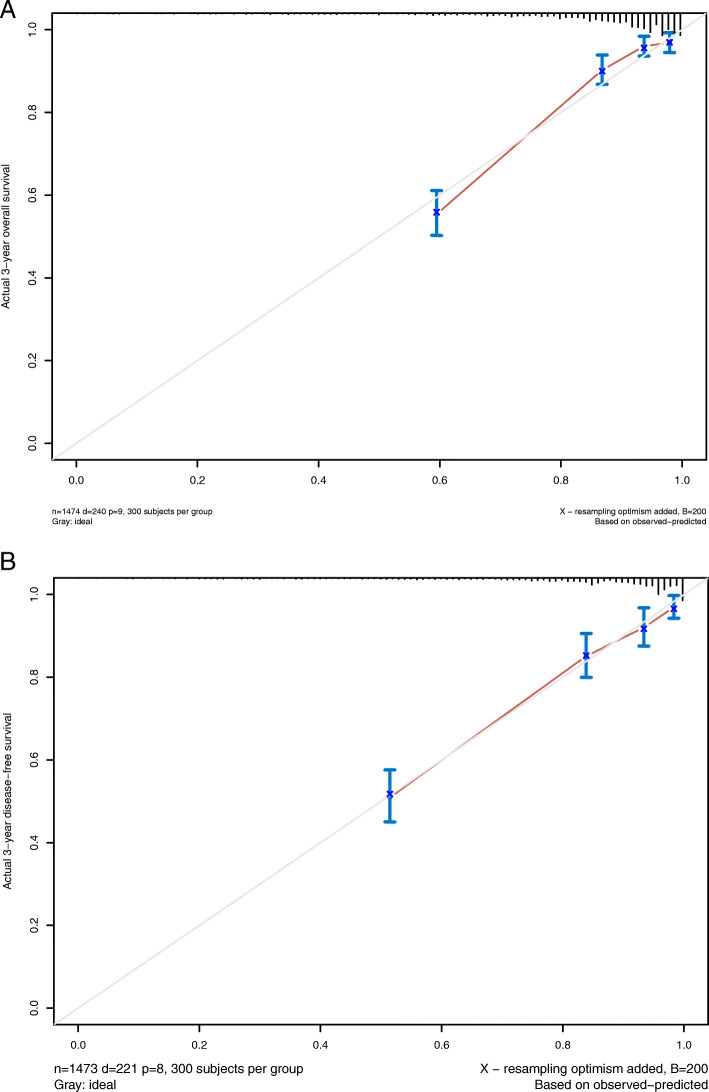
Calibration curves. Calibration curves for 3-year OS (**a**) and 3-year DFS (**b**) using nomograms with clinicopathological characteristics and pretreatment inflammatory biomarkers are shown. The 45-degree reference line represents a perfect match between observed and predicted values

To further evaluate the predictive ability of pretreatment levels of serum bilirubin and TBA in CRC, two nomograms were established by a multivariate Cox regression model based on all the significant independent factors for OS and DFS (Fig. 3a, b). Somke, obstruction, T, N, and M stage, chemotherapy, TBIL, DBIL, and TBA level were included in both prediction models. Nomograms can be created by adding up the scores assigned to each variable, which is indicated at the top of the scale, and the total points can be converted to the predicted 5-year probability of death and recurrence or metastasis for a patient on the lowest scale [11]. Harrell’s C-indexes for OS and DFS prediction were 0.819 (95% CI: 0.806–0.832) and 0.835 (95% CI: 0.822–0.849), respectively, and the calibration curves of the two nomograms showed no deviation from the baseline and no need for recalibration (Fig. 4a, b).

## Discussion

The prognosis of colorectal cancer patients is currently focused on postoperative metastasis and local recurrence of colorectal cancer. At present, there are many studies on prognostic factors in colorectal cancer patients. The primary factors affecting colorectal cancer prognosis include clinicopathology and gene and immune factors [12]. TNM stage, perineural invasion, vascular invasion, chemotherapy, histologic grade, number of resected lymph nodes, lymphatic status, neutrophil-to-lymphocyte ratio and platelet-to-lymphocyte ratio have been confirmed as prognostic factors for colorectal cancer [13, 14]. Other studies have confirmed that colorectal prognosis is closely related to patients’ lifestyle, economic status and educational background, such as low education, inappropriate socioeconomic status, and high tumour grade, smoking, and diabetes mellitus conveyed poor survival of CRC patients [15, 16]. In addition, in this study, we confirmed the prognostic significance of serum TBIL, DBIL and TBA levels in colorectal cancer, revealing that elevated TBIL, DBIL and TBA levels were associated with tumour progression and were independent prognostic factors for colorectal cancer patients. Our nomogram also confirmed the prognostic significance of TBIL, DBIL and TBA in patients with colorectal cancer.

Bile acids are an important component of the gastrointestinal tract. They connect the intestinal microbiota with liver and intestinal metabolism, affecting gastrointestinal motility, intestinal permeability and carcinogenesis. Disruption of bile acid-microbiota crosstalk promotes inflammation and the phenotype of gastrointestinal disease, which may contribute to the development of gastrointestinal cancers, including colorectal cancer and hepatocellular carcinoma [17]. Bile acids are now thought to be involved in the development of cancer, and human epidemiological and animal studies have shown that colon cancer risk is also associated with faecal bile acid concentrations. Abnormally high levels of bile acids trigger excessive harmful effects on the colonic mucosa, such as DNA oxidative damage, inflammation, and proliferation, markedly promoting CRC progression after the initiation phase [18]. Studies have found that bile acid concentration can be used as a prognostic factor in some tumours, primarily because it affects changes in the intestinal flora, leading to the occurrence and development of diseases and contributing to patient prognosis [19]. However, to our knowledge, no studies have reported that levels of total bile acids in patients are an independent prognostic factor in colorectal cancer. In our study, X-Tile software was used to determine the optimal cut-off values of 7.1 μmol/L for TBA for 3-year OS and 6.8 μmol/L for TBA for 3-year DFS. In the clinicopathological characteristics univariate analysis, the high TBA group had a higher percentage of TNM stage (III and IV) than the low TBA group (*P* = 0.007). Multivariate Cox regression analyses confirmed that a high TBA level was an independent prognostic factor for both OS and DFS (*p* < 0.05).

Bile acids are the final product of cholesterol decomposition. They occur in hepatocytes and peripheral blood in three forms: TBIL, DBIL and IDBIL. In addition to total bile acids being considered an independent prognostic factor for colorectal cancer, our results showed that TBIL and DBIL were also independent prognostic factors. The high DBIL group had a higher percentage of obstruction and TNM stage (III and IV) compared to the high DBIL group (*P* = 0.029, *P* = 0.022), and patients in the low DBIL had larger tumour size than those in the high DBIL group (*P* = 0.003). The high TBIL group exhibited a higher percentage of lymph node metastasis (N1 and N2) compared to the low TBIL group (*P* = 0.026), and patients in the low TBIL exhibited larger tumour size than those in the high TBIL group (*P* = 0.008). Multivariate Cox regression analyses confirmed that high DBIL and TBIL levels were independent prognostic factors for both 3-year OS and DFS. At present, similar results have been obtained regarding the prognostic effect of serum bilirubin levels on malignant tumours. Zhang et al. found that high DBIL level was an independent prognostic factor for OS and DFS, and a higher proportion of lymph node metastasis and lymphovascular invasion was observed at higher DBIL levels than at lower DBIL levels [10]. Yang et al. also found that in stage IV CRC patients, elevated levels of TBIL and DBIL were associated with poor OS [20]. DBIL can be considered an independent prognostic biomarker of OS, and the prognostic effect of DBIL on OS is similar to that of carcinoembryonic antigen (CEA). Bilirubin levels have been associated with the risk of several malignancies. Studies have found that moderately elevated pretreatment bilirubin levels are associated with longer OS and DFS in patients with non-small cell lung cancer [21]. A cross-sectional study found that TBIL and DBIL in patients with gastric cancer were significantly lower compared to healthy controls. The effect of bilirubin level on prognosis was similar to CEA and carbohydrate antigen (CA) 19-9 [22]. Sun et al. found that TBIL and albumin levels are independent predictors of OS in patients with gastric cancer, and the combination of TBIL and albumin levels with TNM staging system indicators is of greater prognostic value [23]. Other studies have found that serum bilirubin in patients with advanced pancreatic cancer, TBIL and DBIL are independent of patient OS and are not a prognostic factor [24]. The prognostic significance of serum bilirubin is inconsistent among different types of tumours, and further studies are needed to determine whether pretreatment levels of serum bilirubin is a protective or harmful prognostic factor in CRC.

Although serum markers are an important factor in the prognosis of cancer patients, a single marker may not be sufficient to predict survival in a clinical setting. Combining multiple markers in one index can improve their predictive ability [25–28]. Nomograms combine clinical characteristics to improve the accuracy of survival prediction. In the present study, we constructed a nomogram based on clinicopathological characteristics and pretreatment inflammatory biomarkers (smoking, T stage, N stage, TNM staging, obstruction, TBIL, DBIL, adjuvant chemotherapy) to predict 3-year OS and DFS in CRC patients. Harrell’s C-index confirms the accuracy of these predictions. Harrell’s C-indexes for OS and DFS prediction were 0.819 (95% CI: 0.806–0.832) and 0.835 (95% CI: 0.822–0.849), respectively. These nomograms based on OS and DFS can be used as a practical model for evaluating prognosis in CRC patients.

Our research has some limitations. First, studies have found that the ratio of serum bilirubin to albumin is an important prognostic factor for patients with colorectal cancer with liver metastasis [29]. However, in our study, due to the lack of data from some patients with colorectal cancer with liver metastasis, we did not conduct a separate analysis of these patients, which may lead to errors in the results, and further research will clarify this issue. Second, since a retrospective design was used in this study, there may be confounders affecting the results. Next, this study only analysed the serum levels of bilirubin and bile acid in patients before treatment and was unable to investigate changes in these indicators during treatment or whether these changes affected survival outcomes in CRC patients. Finally, our study was a single-centre retrospective study, and a multicentre study with longer follow-up is needed to verify whether our findings are universally applicable. Nevertheless, several significant advantages of this study include a relatively large retrospective cohort analysis (large sample size) and a broad meta-analysis of various factors that may be associated with colorectal cancer OS and DFS, which should provide an important reference point for clinicians.

## Conclusions

This is the first report to demonstrate the combination of pretreatment levels of serum bilirubin and TBA levels to predict survival outcomes in CRC patients after radical resection in China. Our retrospective study revealed that serum TBIL, DBIL and TBA before treatment were significantly correlated with CRC patient prognosis and were independent prognostic factors. The nomograms based on OS and DFS can be used as a practical model for evaluating the prognosis of CRC patients. This biomarker is directly derived from routine laboratory tests of liver function and can be easily applied in clinical practice. Further study and analysis are needed to investigate its prognostic role in malignant tumours of different organs.

## Data Availability

The datasets used and/or analysed during the current study are available from the corresponding author on reasonable request.

## Abbreviations

CRC: Colorectal cancer
TBA: Total bile acid
TBIL: Total bilirubin
DBIL: Direct bilirubin
OS: Overall survival
DFS: Disease free survival
HR: Hazard ratio
CI: Confidence interval
TNM: Tumor-node-metastasis
AJCC: American Joint Committee on Cancer
CA: Carbohydrate antigen
CEA: Carcinoembryonic antigen
